# Supplementary figures and images for: Sleep-promoting neurons remodel their response properties to calibrate sleep drive with environmental demands
Source: PLoS Biol. 2022 Sep 29;20(9):e3001797. doi: 10.1371/journal.pbio.3001797 (PMC9521806; doi:10.1371/journal.pbio.3001797)

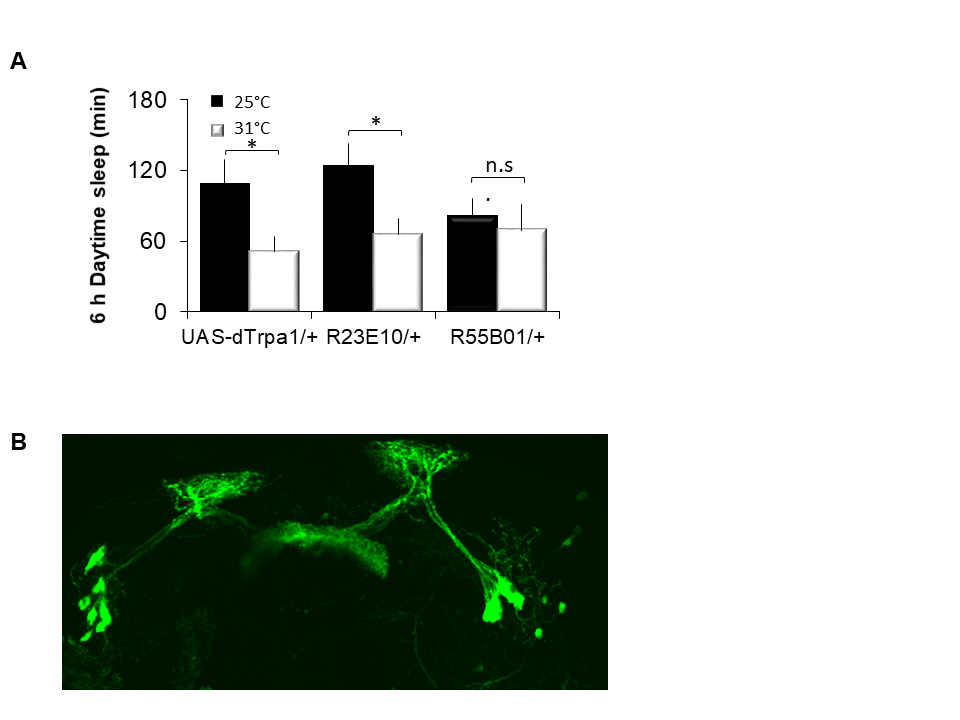

Supplement: S1 Fig — (A) The % change from baseline sleep at 25°C seen following switch to 31°C between 9 AM and 3 PM. (B) Confocal image of R23E10>UAS-Epac1-camps. Underlying data is in S1 Datasheet. (TIF) [file pbio.3001797.s001.tif]

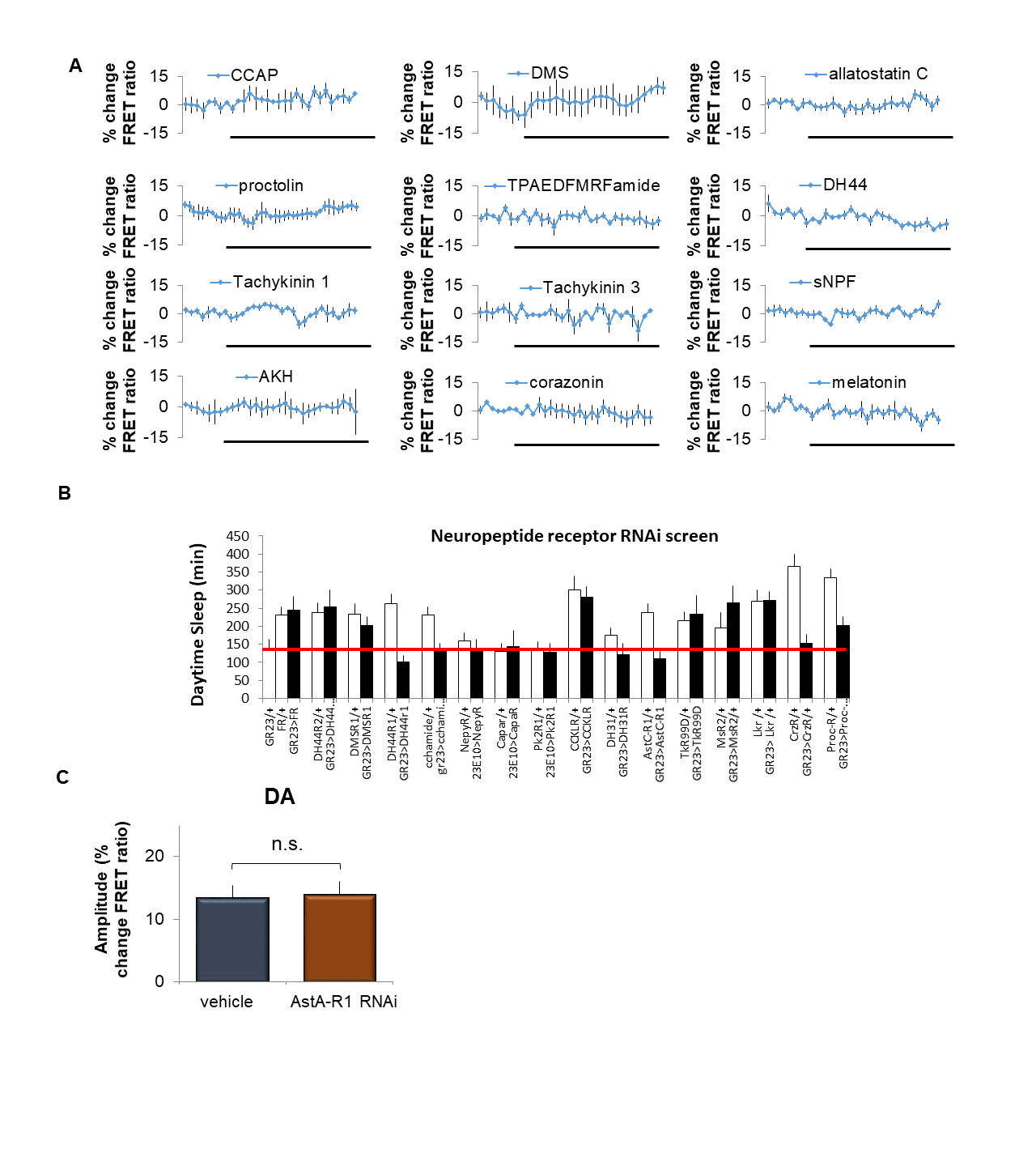

Supplement: S2 Fig — (A) Individual cAMP responses of R23E10 neurons expressing UAS-Epac1-camps shown as % change in FRET ratio during exposure to crustacean cardioactive peptide (CCAP), Drosophila myosuppressin (DMS), allatostatin C (astA C), proctolin, TPAEDFMRFamide, corticotropin-releasing factor-like diuretic hormone 44 (DH44), Tachykinin 1, Tachykinin 3, short neuropeptide F (sNPF), adipokinetic hormone (AKH), corazonin, and melatonin. (B) Daytime sleep in female 5-day-old flies expressing RNAi lines for the depicted neuropeptide receptors using R23E10-GAL4 and their parental controls (n = 14–16 flies/genotype). To be significant, the experimental lines must be significantly different from both the GAL4/+ (red line) and the UAS/+ (white bar) parental controls: FR: FMRFamide Receptor, DHRRR2: Diuretic hormone 44 receptor 2, DMSR1: Myosuppressin receptor 1, DHRRR1: Diuretic hormone 44 receptor 1, cchamideR: CCHamide-1 receptor, NepyR: RYamide receptor, Capar: Capability receptor, Pk2R1: Pyrokinin 2 receptor 1; CCKLR: Cholecystokinin-like receptor at 17D1A, DH31: Diuretic hormone 31,AstC-R1: Allatostatin C receptor 1, TkR99D: Tachykinin-like receptor at 99D, MsR2: Myosuppressin receptor 2, Lkr: Leucokinin receptor, CrzR: Corazonin receptor, Proc: Proctolin receptor. Red line is to facilitate comparisons with R23E10/+ parental control. (C) RNAi knockdown of the AstA-R1 receptor in R23E10 neurons did not affect the cAMP response to DA. Error bars represent SEM. Underlying data is in S1 Datasheet. (TIF) [file pbio.3001797.s002.tif]

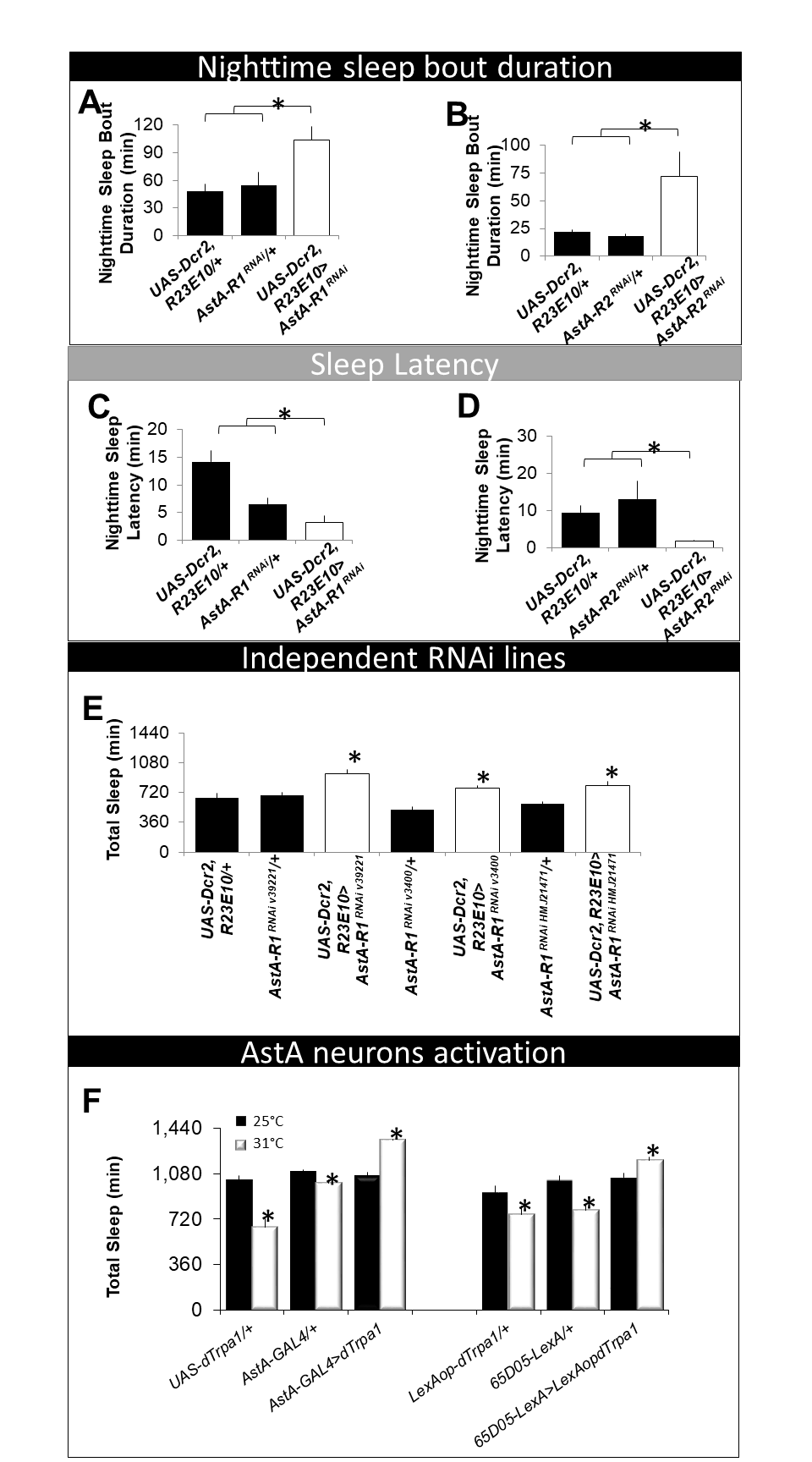

Supplement: S3 Fig — (A, B) Nighttime sleep bout duration is increased in UAS-Dcr2, R23E10-GAL4/+> AstA-R1RNAi/+, and UAS-Dcr2,R23E10-GAL4/+> AstA-R2RNAi/+ experimental flies compared with UAS-Dcr2, R23E10-GAL4/+, AstA-R1RNAi/+, and AstA-R2RNAi/+ parental controls (n = 16/condition, *p < 0.05, modified Bonferroni test). (C, D) Sleep latency is shortened in UAS-Dcr2, R23E10-GAL4/+> AstA-R1RNAi/+, and UAS-Dcr2,R23E10-GAL4/+> AstA-R2RNAi/+ experimental flies compared with UAS-Dcr2, R23E10-GAL4/+, AstA-R1RNAi/+, and AstA-R2RNAi/+ parental controls (n = 16/condition, *p < 0.05, modified Bonferroni test). (E) Total sleep is increased when levels of AstA-R1 is decreased in R23E10 sleep-promoting neurons using 3 additional independent RNAi lines (n = 16/condition, *p < 0.05, modified Bonferroni test). (F) Sleep is increased in AstA-GAL4>UASdTrpA1 and 65D05-LexA>LexAopdTrpA1 flies at 31°C compared with siblings maintained at 25°C; parental controls did not show an increase in sleep at 31°C (n = 14–16/condition and genotype, *p < 0.05, modified Bonferroni test). Error bars represent SEM. Underlying data is in S1 Datasheet. (TIF) [file pbio.3001797.s003.tif]

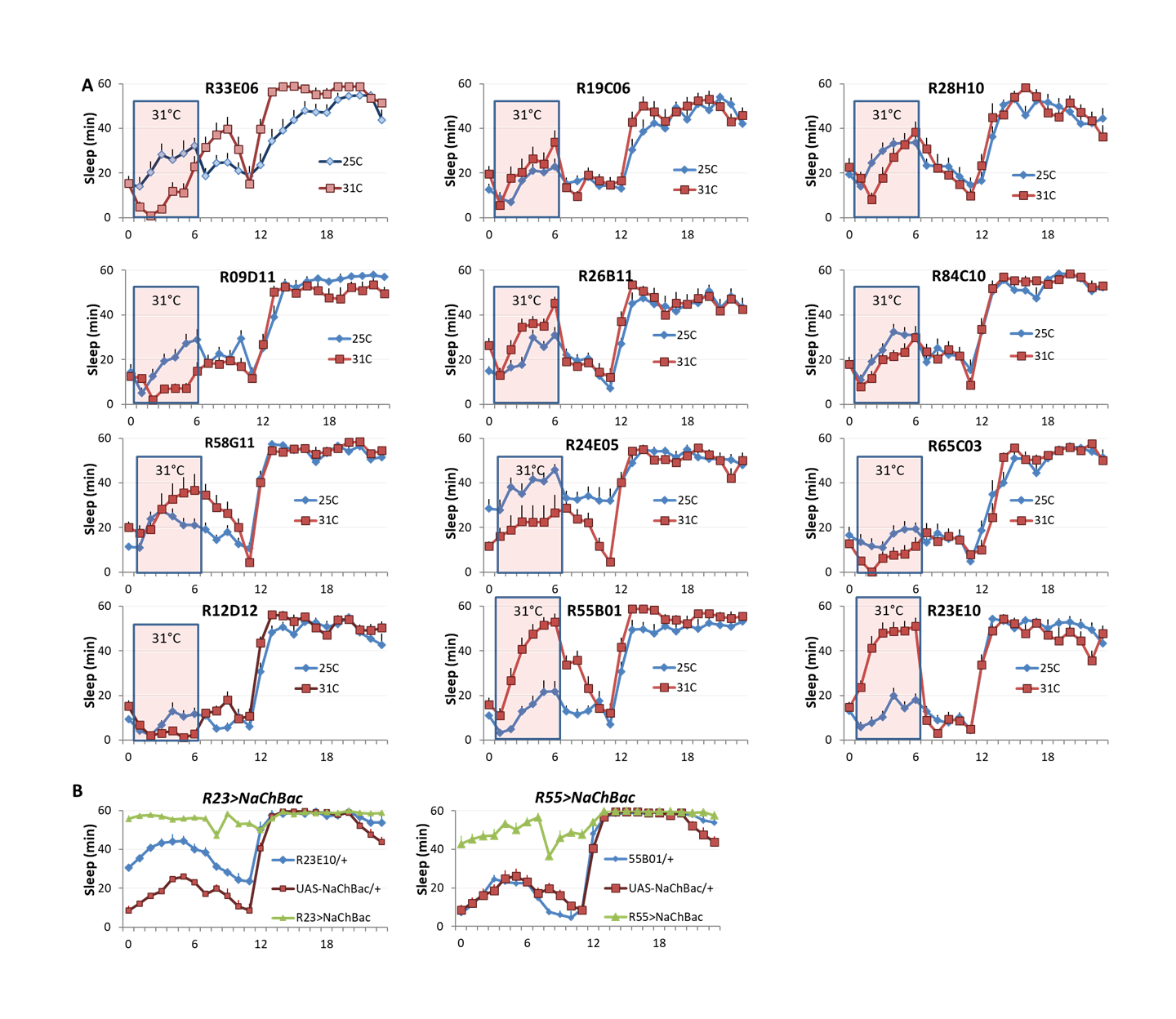

Supplement: S4 Fig — (A) Despite similar anatomical profiles, most dorsal fan-shaped body drivers do not reliably impact sleep when expressing UAS-Transient receptor potential cation channel A1 and raising the temperature to 31°C (n = 14–16 flies/genotype). The data for R55B01>dTrpA are the same as in Fig 3A. (B) R23E10>UAS-NaChBac and R55B01>UAS-NaChBac sleep more than R23E10/+, UAS-NaChBac/+, and R55B01/+ parental controls. Error bars represent SEM. Underlying data is in S1 Datasheet. (TIF) [file pbio.3001797.s004.tif]

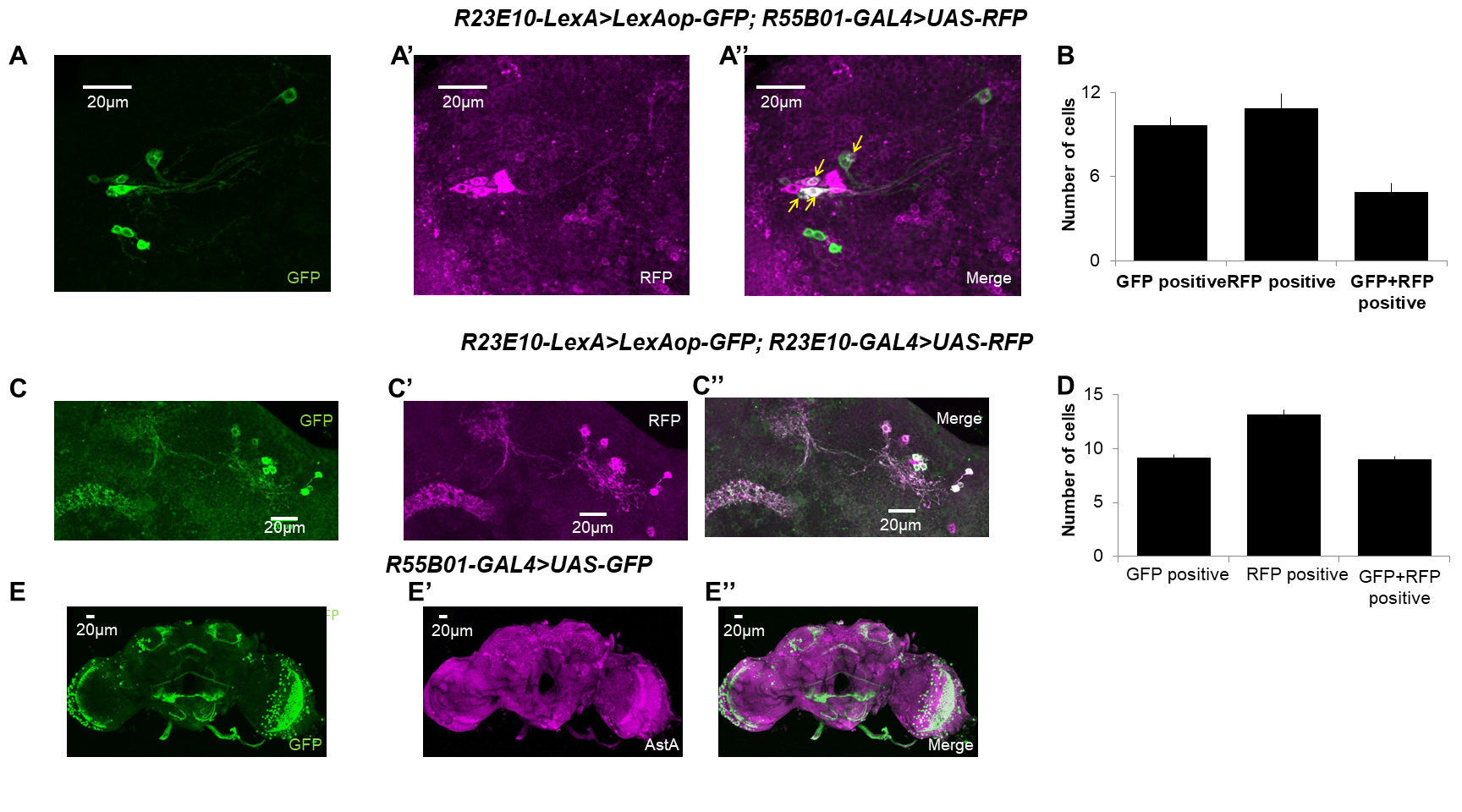

Supplement: S5 Fig — (A) Representative confocal stack focusing on the area containing cell bodies of a R23E10-LexA>LexAop-GFP, R55B01-GAL4>UAS-RFP fly brain stained with anti-GFP antibody, (A’) anti-RFP antibody (magenta) and (A”) a merged image. Yellow arrows on the merge image indicate cells that express both GFP and RFP. (B) Quantification of the number of cells expressing only GFP, only RFP or both GFP and RFP. (C) Representative confocal stack focusing on the area containing the cell bodies of a R23E10-LexA>LexAop-GFP, R23E10-GAL4>UAS-RFP fly brain stained with anti-GFP antibody, (C’) anti-RFP antibody (magenta), and (C”) a merged image. (D) Quantification of the number of cells expressing only GFP, only RFP or both GFP and RFP. (E) Representative confocal stack of a using R55B01-GAL4>UAS-GFP brain stained with anti-GFP antibody, (E’) anti-AstA antibody (magenta), and (E”) a merged image. Error bars represent SEM. Underlying data is in S1 Datasheet. (TIF) [file pbio.3001797.s005.tif]

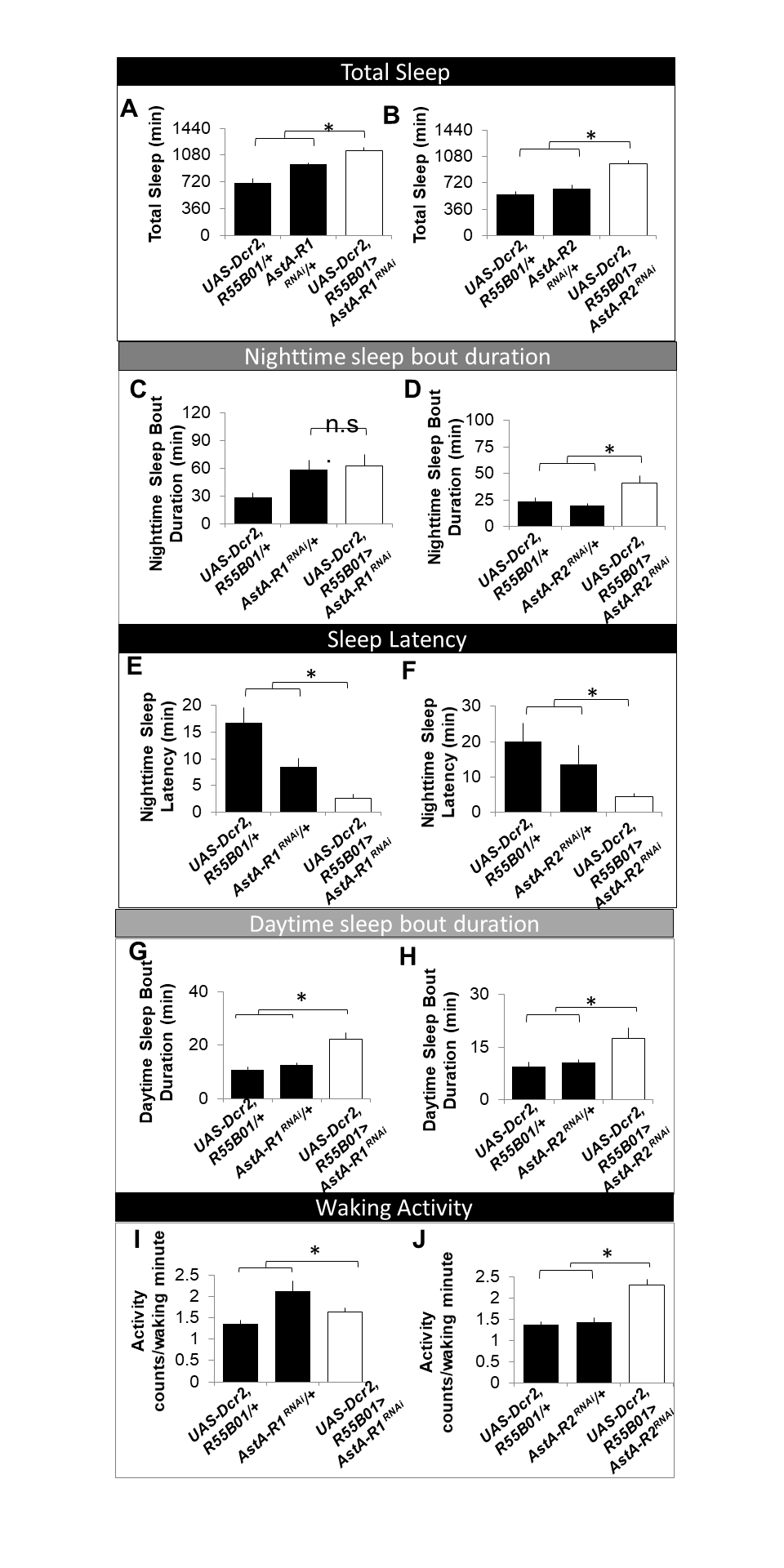

Supplement: S6 Fig — Sleep parameters in UAS-Dcr2, R55B01-GAL4/+> AstA-R1RNAi/+, UAS-Dcr2, R55B01-GAL4/+> AstA-R2RNAi/+ experimental flies and both UAS-Dcr2, R55B01-GAL4/+ and AstA-R1RNAi/+, AstA-R2RNAi/+ control flies for: (A, B) total sleep, (C, D) nighttime sleep bout duration, (E, F) nighttime sleep latency, (G, H) daytime sleep bout duration, and (I, J) counts/waking minute; (n = 16/condition, *p < 0.05, modified Bonferroni test). Error bars represent SEM. Underlying data is in S1 Datasheet. (TIF) [file pbio.3001797.s006.tif]

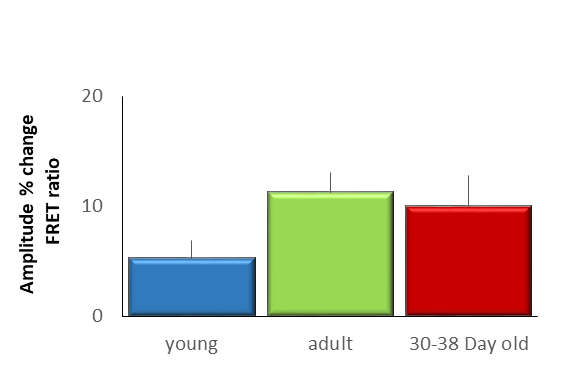

Supplement: S7 Fig — Response of R23E10 neurons in young (0–1 day old), adult (6–8 day old), and old (30–38 day old) flies in response to Dopamine (3e−5 M) (n = 15, 14, 6 cells). The data are expressed as amplitude % change; data for young and adult flies are taken from data in Fig 4B. Error bars represent SEM. Underlying data is in S1 Datasheet. (TIF) [file pbio.3001797.s007.tif]

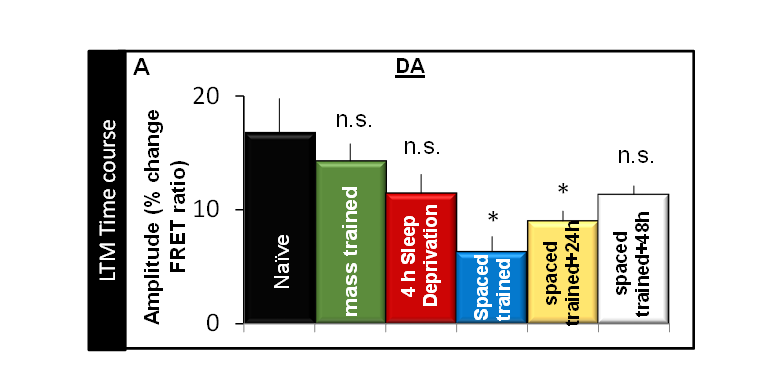

Supplement: S8 Fig — Space-trained flies vs. naïve controls. The reduction of DA responses remained significant 24 h after the end of the training but not 48 h post-training. A Massed training courtship protocol that does not induce LTM had no significant effect on amplitude of DA response in R23E10 neurons; 4 h of sleep deprivation did not alter DA responses in R23E10 neurons (n = 12–25 cells, *p < 0.05). Error bars represent SEM. Underlying data is in S1 Datasheet. (TIF) [file pbio.3001797.s008.tif]

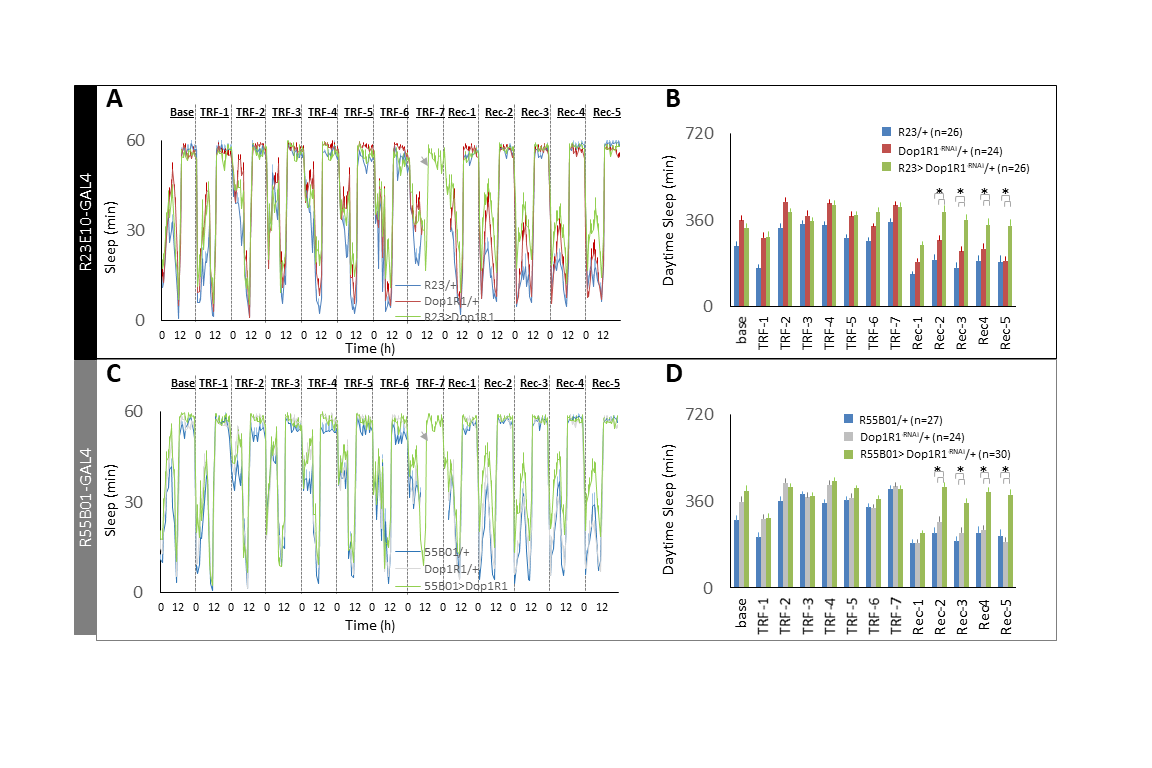

Supplement: S9 Fig — (A) Sleep profiles in R23E10>Dop1R1RNAi, flies and their parental controls R23E10/+, and Dop1R1RNAi/+ during baseline, 7 days of time-restricted feeding and 5 days of recovery (n = 24–26 flies/group); gray arrow indicates disruption in data collection. (B) Daytime sleep is increased following time-restricted feeding in R23E10>Dop1R1RNAi compared to both parental controls. A Genotype (2) x Time (13) ANOVA revealed a Genotype X Time interaction: F[2,24] = 5.5, p = 9.99E-16; *p < 0.05, modified Bonferroni test. (C) Sleep profiles in R55B01>Dop1R1RNAi flies and their parental controls R55B01/+, and Dop1R1RNAi/+ during baseline, 7 days of time-restricted feeding and 5 days of recovery (n = 24–30 flies/group) (Dop1R1-RNAi flies are the same as in A,B); gray arrow indicates disruption in data collection. (D) Daytime sleep is increased following time-restricted feeding in R23E10>Dop1R1RNAi compared to both parental controls. A Genotype (2) x Time (13) ANOVA revealed a Genotype X Time interaction: ANOVA F[2,24] = 7.87, p = 9.99E-16; *p < 0.05, modified Bonferroni test. Underlying data is in S1 Datasheet. (TIF) [file pbio.3001797.s009.tif]

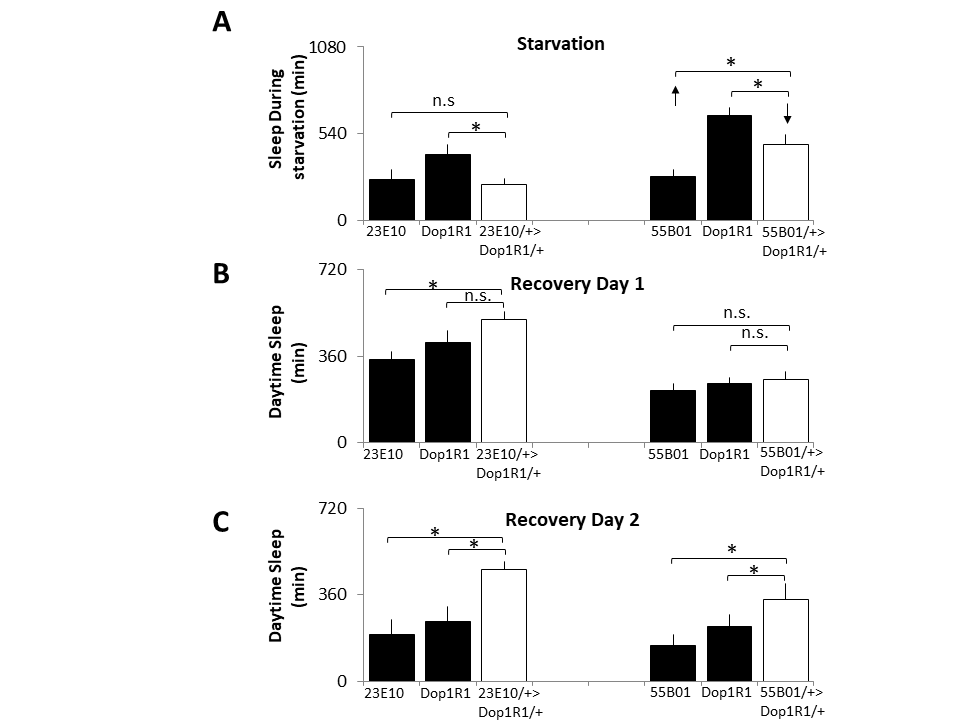

Supplement: S10 Fig — (A) During starvation, sleep in R23E10>Dop1R1RNAi, R55B01>Dop1R1RNAi flies is not consistently above or below R23E10/+, Dop1R1RNAi/+ or R55B01/+ parental controls (n = 13–16 flies/group; ANOVA F[2,36] = 2.2, p = 0.12 and ANOVA F[2,38] = 15.4, p = 1.2E-05, *p < 0.05, modified Bonferroni test). (B) Daytime sleep is not increased in R23E10>Dop1R1RNAi or R55B01>Dop1R1RNAi flies compared to both parental controls R23E10/+, Dop1R1RNAi/+, or R55B01/+ on recovery day 1 (ANOVA F[2,36] = 4.8, p = 0.02 and ANOVA F[2,38] = 0.5, p = 0.58, *p < 0.05, modified Bonferroni test). (C) Sleep is increased in R23E10>Dop1R1RNAi and R55B01>Dop1R1RNAi flies compared to R23E10/+, Dop1R1RNAi/+, or R55B01/+ parental controls on recovery day 2 (ANOVA for Genotype F[2,36] = 14.25, p = 2.75E-05 and ANOVA for Genotype F[2,38] = 8.35, p = 0.0009 for R23E10 and R55B01, respectively. Underlying data is in S1 Datasheet. (TIF) [file pbio.3001797.s010.tif]

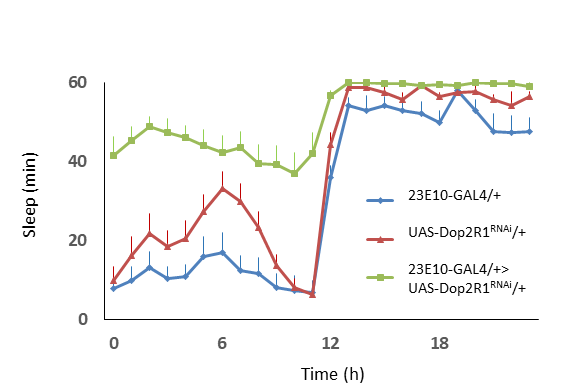

Supplement: S11 Fig — (A) Sleep is increased in R23E10>Dop1R2RNAi, flies compared to parental controls (n = 16 flies/group; 3 (genotype) X 24 (hour) repeated measures ANOVA reveals a significant interaction F[46,1012] = 7.15, p = 0.1.4E-13). Underlying data is in S1 Datasheet. (TIF) [file pbio.3001797.s011.tif]
