# Supplementary material for: Sleep-promoting neurons remodel their response properties to calibrate sleep drive with environmental demands
Source: PLoS Biol. 2022 Sep 29;20(9):e3001797. doi: 10.1371/journal.pbio.3001797 (PMC9521806; doi:10.1371/journal.pbio.3001797)
Supplement: S1 Text — (DOCX) [file pbio.3001797.s020.docx]

**Material and Method**

**Flies**

Unless specified otherwise, only female flies were evaluated. Flies were cultured at 25˚C with 50-60% relative humidity and kept on a diet of yeast, dark corn syrup and agar under a 12-hour light:12-hour dark cycle. *R23E10-GAL4*; *R55B01-GAL4*; *R33E06-GAL4; R19C06-GAL4; R28H10-GAL4; R09D11-GAL4; R26B11-GAL4; R84C10-GAL4; R58G11-GAL4; R24E05-GAL4; R65C03-GAL4*;*R12D12-GAL4* and *LexAop-TrpA1* were a kind gift from Dr Gerald Rubin at Janelia Farms Research campus. *AstA-GAL4*; *R65D05-LexA*; *R23E10-LexA*; *UAS-Dcr2*; *UAS-TrpA1*; *UAS-GFP*; *UAS-RFP, LexAop-GFP*; *AstA-R1^RNAiJF02578^*; *AstA-R2^RNAiJF01955^* ; *Goα47ARNAi HMS01129*; *Giα65ARNAi GL00328*; *Giα65ARNAi HMS01273*; *Gβ5RNAi JF02941*; *Gβ13FRNAi JF01609*; *Gβ76CRNAi JF03127*; *Gγ1RNAi HMS01361* ; *UAS-Dop1R1-^RNAi62193^*; *UAS-Dop1R2^RNAi51423^;* *UAS- FMRFaR ^RNAi 25858^*; *UAS- Dh44-R2 ^RNAi29610^*; *UAS-MsR1^RNAi^* *^27529^*; *UAS-Dh44-R1^RNAi^* *^28780^*: *UAS-*, *cchamideR^RNiI^* *^51168^; UAS-NepyR^RNAi^* *^25944^*; *UAS-CapaR^RNAi^* *^27275^*’ *UAS-Pk2R1^RNAi^* *^29624^*; *UAS-* *CCKLR-17D1^RNAi^* *^67865^*; *UAS-DH31R^RNAi^* *^25925^*: *UAS-AstC-R1^RNAi 27506^*; *UAS-TkR99D^RNAi^* *^27513^*: *UAS-MsR2^RNAi^* *^25832^*; *UAS-Lkr^RNAi^* *^25936^*; *UAS-CrzR^RNAi 26017^*; *UAS-ProcR^RNAi^* *^29414^* were obtained from the Bloomington stock center. *UAS-Epac1.camps* was obtained from Paul Taghert (Washington University in St. Louis). *UAS-P2X2* and *LexAop-Epac1.camps* were a kind gift from Orie Shafer (City University of New York). *AstA-R1^RNAiv39221^* and *AstA-R1^RNAiv3400^* were obtained from the Vienna *Drosophila* RNAi stock center (Austria). *AstA-R1^RNAiHMJ21471^* was obtained from the Kyoto *Drosophila* stock center (Japan). *CaLexA* flies were obtained from Jin Wang (UCSD).

**Sleep**

Sleep was assessed as previously described [52]. Briefly, flies were placed into individual 65 mm tubes containing the same food as they were reared on. All activity was continuously measured through the Trikinetics Drosophila Activity Monitoring System ([www.Trikinetics.com](http://www.Trikinetics.com), Waltham, Ma). Locomotor activity was measured in 1-minute bins and sleep was defined as periods of quiescence lasting at least 5 minutes. All sleep experiments were replicated a minimum of two times.

**Sleep Deprivation**

Sleep deprivation was performed as previously described [50, 73]. Briefly, flies were placed into individual 65 mm tubes and the sleep-nullifying apparatus (SNAP) was used to sleep deprive these flies for 12 hours during the dark phase (lights out to lights on).

**Starvation, Time-Restricted Feeding**

For starvation, flies were individually placed into Trikinetics tubes containing the same food they were reared on and sleep was monitored for 2 days. On the morning of day 3, flies were placed into tubes containing 1% agar and monitored for 18 h. the flies were then placed back into Trikinetics tubes with food. For time-restricted feeding, flies were housed in vials on standard food between 8am and 5pm. At 5pm, flies were transferred to vials containing 1% agar until 8am the next morning when they were transferred to a new vial containing food. Flies underwent this protocol for 7 days. After 7 days, flies were individually placed into Trikinetics tubes containing standard food where they were allowed to eat *ad lib*. Siblings that were maintained in vials with standard food available *ad lib* and flipped at the same times as their restricted counterparts served as treatment controls

**Social Enrichment**

Social enrichment was performed as previously described [51]. Briefly, flies were housed in groups of 20 until 3-4 day old. Then, flies were separated in isolated or enriched groups. Isolated flies were transferred in individual 65 mm tubes. Enriched flies were pooled in groups of 50 flies for 5 days.

**Courtship Conditioning**

Training for naïve males was based on previously described methods [10]. For LTM , a spaced training protocol consisting of three 1-hour training sessions with a mated female, each separated by one hour was employed. For massed training protocol that does not induce LTM, a single 3h pairing with a mated female was employed.

**Live Brain Imaging**

Flies were chilled for approximately 5 minutes prior to pinning them onto a sylgaard dissection dish. Brains were dissected in calcium-free HL3 and then transferred onto a poly-lysine treated dish (35 3 10 mm Falcon polystyrene) containing 3 ml of 1.5mM calcium HL3. Two to four brains were assayed concurrently, typically untreated controls vs a comparison group (e.g. sleep deprived, starved, etc). Image capture was done using an Olympus BX61 and x,y,z stage movements were set via SLIDEBOOK 5.0 (Intelligent Imaging Innovations), which controlled a Prior H105Plan Power Stage through a Prior ProScanII. Multiple YFP/CFP ratio measurements were recorded in sequence from each brain in the dish. Following baseline measurements, 1 ml of saline containing either AstA or DA, was added to the bath (dilution factor of 1/4). We used synthetic AstA (Neo-MPS) and dopamine (Sigma-Aldrich). Experiments were conducted between 8am and 3pm. No differences were observed in responses of either experimental or control brains when experiments were initiated at 8am and compared to experiments ending at 3pm.Crustacean cardioactive peptide (CCAP), drosophila myosuppressin (DMS), allatostatin C (astA C), proctolin, TPAEDFMRFamide, corticotropin-releasing factor-like diuretic hormone 44 (DH44), Tachykinin 1, Tachykinin 3, short neuropeptide F (sNPF), adipokinetic hormone (AKH), corazonin, and melatonin were kindly gifted by Paul Taghert (Washington University in St. Louis). For further details see [42, 43]. For experiment using P2X2, 1 mM ATP was used to activate the P2X2 receptor. ATP was not washed out.

**Immunocytochemistry**

Flies were fixed in 4% PFA, brains were dissected in ice cold PBS and incubated overnight with the following primary antibodies: mouse anti-AstA, (5F10, 1:2 dilution, Hybridoma Bank, University of Iowa), chicken anti-GFP (GFP-1020; 1:1000, Aves Lab), rabbit anti-dsRed (Living Colors DsRed Polyclonal Antibody, 1:250, Clontech). Secondary antibodies were Alexa 488, 594 and 633 conjugated at a dilution 1:200. Brains were mounted on polylysine treated slides in Vectashield H-1000 mounting medium. Confocal stacks were acquired with a 0.5µm slice thickness using an Olympus FV1200 laser scanning confocal microscope and processed using ImageJ.

**Statistics**

All comparisons were done using a Student’s T-test or, if appropriate, ANOVA and subsequent planned comparisons using modified Bonferroni test unless otherwise stated. Note that a significant omnibus-F is not a requirement for conducting planned comparisons [98]. All statistically different groups are defined as *P < 0.05.
