# Supplementary material for: Sleep-promoting neurons remodel their response properties to calibrate sleep drive with environmental demands
Source: PLoS Biol. 2022 Sep 29;20(9):e3001797. doi: 10.1371/journal.pbio.3001797 (PMC9521806; doi:10.1371/journal.pbio.3001797)
Supplement: Attachment — Submitted filename: Response to Reviewers_May 2.docx [file pbio.3001797.s021.docx]

REVIEWS:

Reviewer #1: In this manuscript, Dissel and colleagues dissect the response of sleep-promoting neurons in Drosophila to allatostatin and dopamine, in both normal conditions and under various circumstances that modulate sleep need. They find in particular that dopamine responses are modulated by various starvation paradigms, which also modulate sleep and propose this is due to the upregulation of a specific dopamine receptor that is not normally required for dopamine sensitivity in these neurons under baseline conditions.

The paper is somewhat of a hodge-podge of observation about dFB sleep regulating neurons, spanning allatostatin inputs, a novel sleep-promoting dFB neuron, dopamine inputs, starvation, long-term memory, neural plasticity of receptor expression. This leads to a complicated set of loosely-connected results. However, I am favorably inclined towards this paper, with several interesting observations worthy of publication. I have a few small comments and one bigger comment.

To my mind, the most interesting finding is the sculpting of dopamine receptor subtypes in the dFB neurons and how this alters the integration of sleep responses to starvation. However, the conclusion is based on RNAi behavioral experiments against Dop1R1, showing an impact on sleep in some but not other starvation paradigms (Figure 5+6), combined with cAMP imaging experiments during starvation with RNAi against a different dopamine receptor Dop1R2 (Figure 7), showing that this has no effect on the cAMP signal to dopamine under conditions where starvation-induced sleep is dopamine dependent. The data is consistent with their interpretation, but showing that RNAi against Dop1R1 abrogates the effect would be the most direct demonstration, and I am not clear why this wasn't included.

An increase in sleep is observed after both Time Restricted Feeding (Figure 5) and a night of starvation (Figure 6) when the Dop1R1 is knocked down in R23E10 and R55B01 neurons. That is, both starvation paradigms yield similar outcomes.

R23E10 neurons express Dop1R2 under baseline conditions. As a consequence, knocking down the Dop1R1 will not prevent R23E10 neurons from responding to Dopamine. It is only by knocking down the Dop1R2 and demonstrating that R23E10 neurons no longer respond to Dopamine under baseline conditions that we can evaluate the role of Dop1R1 after starvation.

Other specific comments:

1) the abstract says, "the Drosophila sleep homeostat (dfB)", but this is both confusing and misleading. dFB stands for "dorsal fan-shaped body neurons", not "sleep homeostat" as implied somehow here. More critically, calling these neurons the sleep homeostat is a bit of an overstatement-- the best evidence is that these are an important output arm of the sleep homeostat, but the evidence is out whether the whole sleep homeostatic mechanism is embodied by these neurons.

We agree and have changed the sentence accordingly. The new sentence reads:

“To understand how animals match sleep need with environmental demands, we used live-brain imaging to examine the physiological response properties of the dorsal Fan Shaped Body (dFB) following interventions that modify sleep (sleep deprivation, starvation, time-restricted feeding, memory consolidation).”

2) The P2X2 data showing a connection between AstA-neurons and R23E10 neurons could still be an indirect, or even a long-range, connection, given the slow timescale of the reporter. Does the recently published fly EM map provide any evidence of direct connections?

The fly connectome indicates that there are no direct synaptic connections between LPN-AstA neurons and R23E10 dFB neurons (DOI: 10.7554/eLife.57443 ; doi.org/10.1101/2020.01.21.911859). However, confocal analysis of the anatomy of these cells indicate that their processes are intermingled.

3) Knocking down AstAR2 in R55B01 neurons increases waking activity compared to the control strains. This could suggest an alternative interpretation of a specific sleep effect-- i.e. hyperlocomotion, that should at least be acknowledged in the main text when discussing Figure S6.

In our experience, and with few exceptions, virtually every manipulation that increases sleep in flies also results in an increase in waking activity. In other words, the literature indicates that the increase in waking-activity in long-sleeping flies is not specific to the experimental manipulation that was used to increase sleep. Moreover, the literature also indicates that sleep time is not correlated with locomotor activity (Shaw et al. , 2002).

4) line "we first evaluated the response properties or R23E10 neurons) should read "of"

Thank you, the sentence now reads:

“We first evaluated the response properties or R23E10 neurons of unperturbed flies under conditions characterized by large changes in sleep time.”

5) In the Discussion, comments are made about the data being in conflict with Ni and colleagues, without elaboration. This point should be made clearer-- what was the Ni result specifically, how is your result different?

We appreciate the suggestions. The sentence now reads:

“Although our data appear to be in conflict with that of Ni and colleagues, who provide evidence that AstA-GAL4 provide an excitatory drive onto *R23E10* neurons, we hypothesize that the co-release of inhibitory AstA with excitatory glutamate may attenuate the overexcitement of R23E10 neurons during high sleep drive and allow animals to maintain wakefulness in dangerous or life-threatening conditions.”

6) Discussion: Galanin is mentioned as the homolog of AstA multiple times, when once is enough. It is also a little inaccurate to call it the "mammalian" homolog, when it is in all vertebrates. It also confuses the connection between AstA and Galanin a little bit-- by traditional homology, these peptides look nothing alike. The claim is based on some similarity between the Galanin-receptor and the Allatostatin receptor, but other GPCRs also are similar-- are those orthologs, too? it is fine to claim functional similarities of these inhibitory neuropeptides but let's make the terms of this observation more explicit

We apologize for being imprecise and thank Reviewer #1 for helping us avoid making this mistake. We have modified the sentences throughout the manuscript to state that AstA is functionally similar to Galanin. The first such sentence reads:

“Allatostatin-A is functionally similar to Galanin and has been implicated in both feeding and sleep [27, 37]. “

7) Discussion: "lesioning Galanin neurons in the preoptic area reduces sleep rebound following sleep loss in mice". The impact of galanin loss on sleep rebound was shown first by Reichert 2019 in the Zebra Fish

We thank the reviewer for pointing this out. We now include the citation by Reichert. The sentence reads:

“Indeed, lesioning Galanin neurons in the preoptic area was found to reduce sleep rebound in zebrafish [89] and mice [90].”

8) Conclusion: "Our data reveal that the co-release of AstA is wake-promoting". This is overstated-- the co-release is presumably the hypothesized one with glutamate, but this is not clearly and directly demonstrated in any experiment in this paper.

We agree that we have not provided any evidence for co-release and apologize for the wording. The edited sentence now reads:

“Our data reveal that AstA is wake promoting and likely serves to maintain waking during periods of high sleep drive.”

9) Figure 3A-- the data appears identical to the screen data presented in Figure S4. This should be acknowledged as such, as otherwise it implies the screen result was independently confirmed in a biological replicate, when it does not appear to have been so. It should be done as a separate confirmatory result from the screen in S4.

The reviewer is correct that for reasons of consistency, the data is the same in Figure 3A and in Figure S4. We now note this in the Figure legend for Figure S4.

“The data for 55B01>dTrpA are the same as in Figure 3A.”

I would like to note that the Shaw and van Swinderen labs have worked extensively with the 55B01 GAL4 driver for many years. Activating 55B01 neurons with dTrpA, NaChBac or Chrimson all robustly increase sleep. Other manipulations such as knocking down AstAR1, AstAR2, Dop1R1 etc. also increase sleep.

10) Figure 4 lacks stats, which are buried in the Figure S7, but these are critical for the interpretation of the curves. Couldn't a repeated measures stat capture this in the same panel? Or perhaps the graphs of the supplement can be made into smaller insets, especially for the key findings?

We thank Reviewer #1 for the suggestion of adding the data from Figure S7 to Figure 4 as insets. We have now done so and believe that this modification makes the data much more accessible.

11) Figure 7D, the 55801/+>Epac/+ bar is small and slightly negative, but this doesn't quite match the data shown in Figure 7C, which has a short dip followed by data that are mostly positive. This disconnect makes me concerned about how the time series are being normalized and summed/averaged to generate the bar graphs that form the basis of the data analysis, here and in other figures measuring the cAMP levels. Would a time-series repeated measures stat serve better here?

We calculate the peak response by averaging the data from three time points immediately after the application of the ligand. This approach is standard in the field. It important to note that the data in the bar graph always accurately reflect the data shown in the line graph. Regardless of how the data is quantified, the data in Figure 7 clearly demonstrate that neurons show a larger response to dopamine during recovery from starvation.

12) In general, the standard for depicting data in figures nowadays is to show all the data points, not just a bar graph with SEM. That allows for a fuller representation of the data, highlights the Ns and variance involved, etc. I would highly recommend and strongly prefer such plots for all the bar graph data in the manuscript. e.g. Kempf et al., 2019. or Grubbs et al 2020 for a sleep-paper model systems in PloS Biology example.

We acknowledge that there has been a trend for including all data points in the figure. I find this trend to be distracting and confusing. However, I believe that access to the underlying data is critical. As a consequence, we will provide all of the individual data points for each figure in supplemental files.

Reviewer #2: The authors examined the role of allatostatin-A (AstA) on sleep-promoting R23B10/R55B01 positive dorsal fan-shaped body (dFB) neurons of Drosophila and found AstA decreased intracellular cyclic AMP (cAMP) level (Fig 1). Since knocking down of either AstA-R1 or AstA-R2 increased sleep (Fig 2, Fig 3GD), they proposed AstA promotes wakefulness by inhibiting dFB neurons, although the activation of AstA positive neurons by AstA-GAL4>dTRP-A1 promoted sleep (Fig S3C). They also examined the changes in the cAMP responses of R23B10/R55B01 positive dFB neurons to AstA and dopamine (DA) under different conditions, which affect sleep behaviors (Fig 4). Various changes were observed and apparently the responses to AstA and DA were independently and context-dependently controlled.

The authors also found that knocking down of Dop1R1 in R23B10/R55B01 positive dFB neurons, which did not affect sleep amount under ordinary condition, increased sleep under restricted feeding protocol (Fig 5). Knocking down of DopR1 in dFB also changed the sleep behavior during the recovery period from starvation (Fig 6). They showed the responses of dFB neurons with Dop1R2 knockdown to DA were changed from starvation to recovery (Fig 7).

With all these data, the authors concluded that dFB neurons changed their state, i.e. responsiveness to AstA and DA, which may be the basis for the distinction of the different type of sleep needs.

I enjoyed reading the manuscript and found it interesting and informative, although there are some inconsistency and insufficiency.

Major questions.

1. The biggest changes in sleep need and arousal threshold occur between day and night. I think it is necessary to examine the changes of dFB neurons at different time of the day. At least, the time of the experiments should be clearly stated.

Data were collected during the light period between 8am and 3pm. No differences were observed between experiments initiated at 8am and those ending at 3pm. This is now clearly stated in the Method section.

With respect to time-of-day effects, the data presented in figure 6 does indeed suggest clock regulation of these circuits. Such an observation is consistent with the literature (DOI: 10.1371/journal.pgen.1006492) and is extremely interesting to us. However, this aspect of sleep regulation is outside the scope of the current study and is being actively investigated as an independent story.

2. Were the experiments of Fig 4 and Fig 7 performed with TTX? Otherwise, it is possible that the difference in the intrinsic release of AstA and DA modified the response of dFB neurons to them. This possibility should be clearly stated.

We did not use TTX for the datasets in Figure 4 or Figure 7. We thank the reviewer for this suggestion. We have now added a sentence to the Discussion section to highlight this possibility. The new sentence reads:

“It should be emphasized that we did not use TTX when evaluating the response properties of *R23E10* neurons to different types of waking. As a consequence, it is possible that the difference in the intrinsic release of AstA and DA during these manipulations modified the response of dFB neurons. Such a possibility will be evaluated in future studies.

3. In addition to above, although this might be technically difficult, the basic intracellular cAMP level of dFB neurons before the application of AstA and DA may be different according to the context in Fig 4. This difference affects the magnitude of AstA and DA responses differently, since the direction of the response is reverse, i.e. decrease and increase. This should be also discussed.

The baseline CFP/YFP ratios that we see in ex-vivo preparations prior to application of ligand are similar and thus cannot explain the differences in DA or AstA amplitude observed following the experimental manipulations.

4. The interpretation of Fig 6 is too speculative. Since there is no change in DA response after starvation in control flies as shown in Fig 4h, the mechanism would be different from Dop1R1 induction. The authors should demonstrate actually induction of Dop1R1 by immunohistochemistry or other methods. Fig 7 is also pretty incomplete. Since, there is no change in the DA response of the control fly after starvation, the DA response change in the flies during recovery period and that of the Dop1R2 RNAi flies after starvation should be included. In addition, since the magnitude of the effect of time restricted feeding in Fig 5 is very big, it is better to include the live imaging data of Dop1R1 knockdown before and after TRF.

Figure 4 and Figure 7 depict very different types of experiments. In Figure 4, the *R23E10* neurons were evaluated in flies during starvation (flies were not given time to recovery). In contrast, in Figure 7, the response of *R23E10* neurons were examined after two days of recovery. This is an important point and highlights that the recruitment of the Dop1R1 does not occur during starvation. The response of R23E10 and 55B01 neurons during recovery from starvation is shown for controls in Figure 7a,c .

We disagree that time-restricted feeding experiment is superior to evaluating flies during recovery from a single and acute episode of starvation.

In our experience, immunohistochemical identification of Dop1R1 is imprecise due to high background noise which is particularly problematic for quantifying receptors in fine processes. In addition, the receptors might be present but not inserted in the membrane. As a consequence, the immunohistochemistry doesn’t answer the question directly as one might hope.

Minor points

1. Please state the sex of the flies used clearly, although the authors wrote there were no big sexual dimorphism.

We agree that this is an important point. We now state at the beginning of the method section:

“Unless specified otherwise, only female flies were evaluated.”

2. Please include the more discussion about Fig 4. For example, I do not understand why the response to DA did not change after sleep deprivation in Fig 4d.

To date, the assessment of dFB neurons following sleep loss has relied almost exclusively on electrophysiology. In contrast, our data has examined how sleep deprivation alters the coupling of dopamine receptors to cAMP. The most parsimonious interpretation of the data is that sleep deprivation does not change the amplitude of the response to cAMP. Rather, the continual activation of Dop1R2 during 12 h of sleep loss would lead to a sustained change in cAMP over time, which would then change the physiological response properties of dFB neurons through the downregulation of Shaker and the upregulation of voltage-independent leak currents via Sandman.

3. The abstract should include Ast-A and DA which is helpful to the authors.

We thank the reviewer for this suggestion. We have modified the abstract. The new sentence reads:

“We report that dFB neurons can distinguish between different types of waking and can change their physiological response-properties to Dopamine (DA) and Allatostatin-A (AstA) accordingly.”

4. The phrase "Rather, the dFB neurons themselves can determine (modulate) their response to the activity from upstream circuits" in the abstract does not make sense to me. I think the authors showed that dFB neurons changes their responsiveness from the previous inputs to them from other neurons, but not they sense the sleep need for themselves.

We have modified the abstract. This section now reads:

“We report that dFB neurons change their physiological response-properties to Dopamine (DA) and Allatostatin-A (AstA) in response to different types of waking. That is, dFB neurons are not simply passive components of a hard-wired circuit. Rather, the dFB neurons intrinsically regulate their response to the activity from upstream circuits..”

Reviewer #3: In this manuscript, Dissel et al examine examine how the dFB and input neurons are regulated in response to a number of different stresses. The manuscript focuses on two neuromodulations, AstA and DA, that impact R23E10 neurons that innervate the dFB. A major strength of this manuscript is that it provides cellular-level resolution of how sleep neurons are regulating depending on experience. This aspect of the paper is likely to generate broad interest because conveys the need to understand mechanisms regulating sleep in a number of different conditions. A number of experimental concerns are described below, some of which can be addressed through written clarification). There is a heavy reliance on ex vivo preparations, which may not reflect the brain function in an intact animal. In addition, the manuscript is written in a descriptive manner, and streamlining the writing and figures may help to convey the conceptual advances and increase the impact of the manuscript.

1. I find the logic laid out in the abstract suggesting the dFB determine responses to be confusing. The way it is written, it seems to confer cognitive capacity on a set of neurons. The data suggests these neurons are integrators of information of other areas, impacting the behavioral output. In the third paragraph of the introduction the logic is laid out, but it is not clear what the alternative is to being impacted by upstream regulators.

As mentioned above, we have changed the abstract to reduce the interpretation that neurons have cognitive capacity. In this case we intend to emphasize that dFB neurons sculpt which receptors they express depending upon their prior history. Thus, the dFB neurons are not simply responding passively to incoming signals. We are open to further modifying this description upon request.

2. Why does Epac1-camps label fewer cells than GFP? Are the eight a different subset? Is the difference due to sensitivity/different levels of R23E10? It would be useful to show these neurons.

We interpret this result as being due to lower expression of UAS-Epac in some neurons such that the signal is too low to see. We now include a confocal image of R23>UAS-Epac1-Camps in Supplemental Figure 1B.

3. In 1i, Asta1-RNAi nearly abolishes FRET, but both AstARs impact sleep in Figure 2. Please explain and/or include the effects of AstA-R2-RNAi on Epac1 FRET.

Our data indicate that the dFB represent a heterogeneous group of neurons. Moreover, UAS-Epac1-Camps does not label all neurons represented in the expression pattern of either R23E10-GAL4 or 55B01-GAL4. In addition, the data presented in Figure 1h,i indicates that knocking down AstAR1 only attenuates the response of R23E10 neurons to AstA but that R23E10 neurons still respond (the % change in FRET ratio is ~-7%). Critically, within the dFB, gap junctions play an important role in sleep regulation (DOI: 10.7554/eLife.37105). Finally, we should note that we are only monitoring cAMP from cell bodies and not processes. Recent studies suggest that, at least for some peptidergic neurons, there can be a differentially regulated release of peptide at the soma and terminals (DOI: 10.1073/pnas.2101818118).

4. If data were acquired where ATP is washed out in 1L this is important to show.

We now state that ATP was not washed out in the Method section.

5. Fig 2I does not fully test the hypothesis that AstA signals through the identified G-proteins because there are likely many receptors using G-proteins in those cells. I suggest toning down this statement. Also, it's not clear what the alternative mode of action would be for AstaRs since they are G-protein coupled.

We thank the reviewer for this comment and agree entirely. We have now modified this sentence to more accurately reflect the experiment. The new sentence reads:

“Thus, these data suggest that the inhibition of dFB neurons can be achieved via coupling with inhibitory G proteins.”

6. There is a large reliance on ex vivo imaging through. It is important to highlight the limitations of this or validate with an in vivo preparation. In addition, given the role of AstA in circadian regulation, emphasizing the timing of experiments throughout the manuscript would be helpful.

We now include a statement in the Method section noting when the experiments were conducted. The sentences read:

“Experiments were conducted between 8am and 3pm. No differences were observed in responses of either experimental or control brains when experiments were initiated at 8am and compared to experiments ending at 3pm.”

We believe that the readership is very much aware of the strengths and weakness that are present in both ex-vivo and in-vivo experiments. Ex vivo imaging is a proven tool that has been used effectively by neuroscientists in a large variety of fields for many decades. Indeed, the use of ex-vivo imaging has been so well validated and discussed over the last half century that it really needs no additional comment here. Importantly, our labs conduct both ex-vivo and in-vivo studies. As a consequence, we do not want to throw rocks at glass houses. Like ex-vivo studies, in vivo studies also suffer from a variety of confounds, especially when evaluating sleep. As mentioned, we believe that our readers are aware of the benefits and limitations of the tools available and can make their own judgments.

7. A weakness is the focus on AstA and DA, but there are likely additional contributors to the regulation of R23E10 neurons.

Most neurons in invertebrate and vertebrate brains respond to a surprisingly large variety of neurotransmitters, neuropeptides and neuroactive compounds (lactate adenosine etc). R23E10 neurons are no exception. Indeed, the literature is abundantly clear that other factors contribute to the activity of R23E10 neurons. For example, the literature supports a role for Glutamate (DOI: 10.7554/eLife.40487), GABA (DOI: 10.1038/s42003-020-0902-8), antimicrobial peptides (DOI: 10.1126/science.aat1650), 5HT2b receptor (DOI: 10.7554/eLife.26519), gap junctions (DOI: 10.7554/eLife.37105) amongst others. Nonetheless, many labs are particularly interested in AstA and DA. Given our interest in AstA and DA coupled with the large number of experimental manipulations employed here, the focus on two ligands seems reasonable. Future studies in many labs will evaluate other factors and how they might interact to regulate sleep.

8a. The TRF protocol used in figure 5 lists the restriction from 8AM-5PM. This should be in ZT, but it is also unclear why this timeframe was chosen, since it does not match the light dark cycle or any obvious component of the sleep cycle.

We thank Reviewer 3 for noticing that we did not use ZT in reference to the TR protocol; this has now been fixed.

We chose the 8am-5pm time window based upon discussion with Professor Satchin Panda at the 2018 Sleep Regulation and Function Gordon Research Conference. Indeed, the literature is increasingly clear that the effectiveness of time-restricted feeding in a variety of animals can be achieved over a surprisingly large time-window as long as it includes night-time fasting. Interesting, a recent publication used a trial and error approach to develop a protocol that was able to impact lifespan (DOI: 10.1038/s41586-021-03934-0). The protocol we describe did not require fine-tuning or optimizing. Importantly, we show this protocol to be effective. An added feature is that the protocol is simple and straightforward. Importantly, it can easily be adopted by a variety of labs without inducing an undue burden on lab personnel.

8b. I also question the idea that TRF results in substantial metabolic challenge. An alternative way of interpreting TRF is that misalignment is a metabolic challenge, and the metabolic stress is reduced during TRF.

We thank Reviewer #3 for pointing out that TRF may not actually be a substantial metabolic challenge. We agree and have modified the sentence; it now reads:

“Since 18 h of starvation did not change the response properties of R23E10 neurons (Figures 4G,H), we used time-restricted feeding to safely impose as an alternate challenge of longer duration”

8c. The robustness of the effects in 5F-I is very compelling. It would be informative to have data on sleep during the TRF protocol.

The Time Restricted Feeding protocol in which flies are rapidly and gently swapped between vials, is extremely robust. In contrast, repeatedly swapping individual flies between small glass Trikinetics tubes is not gentle, takes a fairly long time and seems to activate arousal centers. In our standard sleep recording protocols, we never evaluate sleep in flies on the day that they are placed into tubes for these very reasons.

With that in mind, we evaluated sleep during Time Restricted Feeding as requested by Reviewer #3 (see Figure S7 below). Individual flies were transferred from glass-tubes with food to glass-tubes containing 1% agar at 5pm and then transferred back to tubes containing food at 8am. In all we conducted 4 independent replicates using R23E10>Dop1R1-RNAi, R55B01>Dop1R1-RNAi and their parental controls (R23E10/+, R55B01/+ and Dop1R1/+). In each replicate, sleep was increased when flies were released into recovery; a representative replicate is shown below in Figure S7). However, the changes in sleep that we observed during the course of time-restricted feeding varied between replicates. In some replicates R23E10>Dop1R1-RNAi and R55B01>Dop1R1-RNAi showed progressive increases in daytime sleep during Time Restricted Feeding. In other replicates no changes in daytime sleep were found. Although it may eventually be possible to optimize this protocol to reduce confounding variables, we would not recommend that other labs transfer individual flies to evaluate sleep during Time Restricted Feeding. However, we have replicated the original observation that Time Restricted Feeding leads to an increase in sleep in *R23E10>Dop1R1-RNAi* and *R55B01>Dop1R1-RNAi* flies even when flies experience prolonged handling.

9. This may be semantic, but I think it's conceptually important to lay out whether Dop1R1 'supports waking behavior during recovery from starvation' or inhibits homeostatic recovery.

Our data favors the interpretation that Dop1R1 supports waking during recovery from starvation. Specifically, sleep homeostasis is most robustly observed in the first several hours immediately after sleep loss. In fact, the vast majority of fly sleep labs only record sleep homeostasis measured as the increase in sleep for the immediate 6 h during recovery. In contrast to sleep homeostasis, the changes in sleep in R23E10>Dop1R1-RNAi and R55B01>Dop1R1-RNAi flies are not robustly observed until ~24 h after the flies have been placed back onto food.

Minor:

1. In the abstract, the (dFB) abbreviation is confusing because it comes after the sleep homeostat, but is not an abbreviation for the homeostat.

We agree and have modified the sentence, it now reads:

“To understand how animals match sleep need with environmental demands, we used live-brain imaging to examine the physiological response properties of the dorsal Fan Shaped Body (dFB) following interventions that modify sleep (sleep deprivation, starvation, time-restricted feeding, memory consolidation).”

2. The abstract discusses dFB, but the introduction describes R23E10 neurons, and then the results describe R23E10 ad projecting onto the dFB. Clearly laying out the circuit in the abstract/into would be helpful.

We apologize for the confusion. The neurons are categorized as dFB based upon their expression pattern. We do not know how to make this explicit in the abstract without breaching the word limit or sacrificing other critical details.

3. I find this sentence awkward. It would seem proper to thank them in the acknowledgments rather than describing the request in the results section. 'To expedite the identification of such lines, we asked Dr. Gerry Rubin and Dr. Arnim Jenett, for assistance [49]'

We have changed the sentence to read:

“We obtained 12 GAL4 lines the Flylight collection that were selected to match the expression pattern of the original sleep promoting GAL4 lines (104y and C5) [10, 49].”

We now acknowledge Drs Rubin and Jenett in the acknowledgements.

I would like to emphasize that Drs Rubin and Jenett put a fair amount of time into identifying the drivers for us. Their involvement was not casual.

4. Check to make sure the Epac transgene is consistently written throughout the manuscript text and figures

We thank the reviewer for noticing this incongruence and now use the UAS-Epac1-camps consistently.

5. In discussion, suggest changing the phrase 'historical context' to something more specific.

We thank Reviewer #3 for this suggestion. We have removed the term “historical context” and now refer to the experiments conducted.
