# Supplementary material for: Sleep-promoting neurons remodel their response properties to calibrate sleep drive with environmental demands
Source: PLoS Biol. 2022 Sep 29;20(9):e3001797. doi: 10.1371/journal.pbio.3001797 (PMC9521806; doi:10.1371/journal.pbio.3001797)
Supplement: Attachment — Submitted filename: Response_Shaw.docx [file pbio.3001797.s022.docx]

Lucas Smith, Ph.D.

Dear Dr. Smith,

We found Reviewer #1’s initial comments to be very insightful and extremely helpful. However, despite Reviewer #1’s obvious expertise, we feel that their single remaining concern may be the result of confusion due to the organization of the manuscript. If possible, we would like to discuss this point of confusion with you and the academic editor. Our goal is to help clarify the precise nature of the concern.

As outlined below, we have several questions about the relevance of evaluating the impact of starvation on the response of *R23;UAS-Epac>Dop1R1^-RNAi^* neurons to dopamine during the recovery phase following starvation.

The first question is whether we have provided genetic evidence to support the interpretation that knocking down the *Dop1R1* in *R23* and *R55* neurons is sufficient to increase sleep following starvation and time restricted feeding (Figures 5 and 6). The answer to this question is important. If the answer is affirmative, a new question arises: Can we conclude that the *Dop1R1* does not play a role in sleep regulation if ex-vivo imaging in *R23;Epac>Dop1R1^-RNAi^* neurons show any cAMP response to the application of dopamine? To be precise, we do not understand how direct genetic evidence linking the *Dop1R1* in *R23* and *R55* neurons to behavior (Figure 5 and 6) can be refuted by a physiological outcome measured using an ex-vivo preparation.

A related question, is whether an ex-vivo imaging study is able to tightly link behavior and neurophysiology. Our reading of the literature indicates that the very nature of the ex-vivo prep makes it impossible to directly link an outcome to behavior. Indeed, interpretation of ex-vivo neurophysiological changes are typically verified by specific genetic behavioral follow up studies. Indeed, not all neurophysiological changes are accompanied by changes in behavior. A recent paper in J Neuroscience (DOI: [10.1523/JNEUROSCI.1488-20.2020](https://doi.org/10.1523/jneurosci.1488-20.2020)) shows that physiological changes observed when knocking down various dopamine receptors does not always result in the predicted changes in sleep. Also note that initial papers evaluating the dorsal Fan Shaped Body concluded that the *Dop1R1* played a role in the dorsal Fan Shaped Body based upon physiological data but without genetic evidence (DOI: [10.1038/nn.3238](https://doi.org/10.1038/nn.3238) and DOI: [10.1016/j.cub.2012.09.008](https://doi.org/10.1016/j.cub.2012.09.008)). Each of these three examples emphasize that physiological experiments are not deterministic and are best used in combination with direct tests of behavior. As seen in Figure 5, 6 and 7 we take this approach in our manuscript.

It seems to us that studies similar to ours tend to be formatted to show the correlational physiological imaging study first. Genetic studies are then shown in subsequent figures to determine if the predicted behavioral outcome is observed. We would be happy to switch the order of the behavioral-genetic studies (Figures 5 and 6) and the ex-vivo imaging study (Figure 7) if it helps the readers follow the story more easily and to emphasize that the conclusion about the role of the *Dop1R1* in *R23* and *R55* neurons in regulating sleep can only be supported by direct genetic studies focused on behavior itself.

Finally, we do not understand how examining the cAMP response of *R23;UAS-Epac>Dop1R1^-RNAi^* to dopamine can provide additional clarity because *R23* neurons respond to dopamine via the *Dop1R2*. As mentioned in our original submission, we have replicated previous reports demonstrating that *Dop1R2* operates in *R23* neurons to regulate sleep (DOI: 10.1038/nature19055). As seen in Figure R1 A,B, *R23>Dop1R2^RNAi^* flies sleep more than parental controls during baseline, and these changes in sleep persist following starvation. Thus, *R23;UAS-Epac>Dop1R1^-RNAi^* must respond to dopamine after starvation because the *Dop1R2* is still present in *R23* neurons.

We have also knocked down *DopEcR* in *R23* and *R55* neurons and evaluated sleep during baseline and following starvation. *DopEcR^-RNAi^* modulates sleep during starvation (Figure R1 C,D and data not shown). We have chosen not to include *DopEcR* data in the current manuscript because we do not observe the change in sleep during recovery after each replication. That is, while *R23>DopEcR^-RNAi^* flies routinely show increases in sleep after starvation, it is also common for sleep to remain similar to parental controls. Unfortunately, despite over 2 years of vigorous investigation, we have not been able to identify the source of variability between replicates. Since we cannot consistently reproduce sleep phenotypes during recovery from starvation when using *DopEcr* we do not wish to include the data in the manuscript. However, our reading of the literature indicates that all 4 DA receptors can alter cAMP in neurons exposed to dopamine (DOI: [10.1523/JNEUROSCI.1488-20.2020](https://doi.org/10.1523/jneurosci.1488-20.2020), DOI: [10.1371/journal.pgen.1003843](https://doi.org/10.1371/journal.pgen.1003843), DOI: [10.1371/journal.pgen.1003843](http://dx.doi.org/10.1371/journal.pgen.1003843), DOI: 10.1523/JNEUROSCI.1005-05.2005). Thus, we cannot exclude the possibility suggested by Reviewer #1 that the dopamine signal observed in Figure 7 can be due to another source under all circumstances. However, we are certain that that knocking down the *Dop1R1* in *R23* and *R55* neurons is sufficient to increase sleep following starvation and time restricted feeding (Figures 5 and 6). Importantly, we state very clearly that we cannot exclude the role of other dopamine receptors when focused solely on ex-vivo imaging. However, our conclusions are measured and accurate: “While we cannot exclude a role of other Dopamine receptors (e.g., Dopamine/Ecdysteroid receptor) for the observed changes, when viewed with the sleep experiments shown above (Figures 5 and 6), these data suggest that during recovery from starvation the constellation of Dopamine receptors in *R23E10* and *R55B01* changes and most likely includes the recruitment of *Dop1R1*.”

Although the tools are available to knock down dopamine receptors in various combinations, we do not believe that ex-vivo imaging data can alter the main conclusions of the paper and, in addition, will create additional questions that are not experimentally tractable. We will conduct the proposed experiment if necessary but would prefer not to delay the publication of the paper if at all possible. Your guidance in this regard is very much appreciated.

Paul
